# Early evaluation of a DBT-informed online intervention for people with eating disorders

**DOI:** 10.1186/s40337-024-00974-5

**Published:** 2024-01-19

**Authors:** Laura Vuillier, M. Greville-Harris, C. V. Talbot, L. May, R. L. Moseley

**Affiliations:** 1 Department of Psychology, Bournemouth University, Poole, UK; 2 Southern Health University NHS Foundation Trust, Southampton, UK

**Keywords:** Eating disorders, Alexithymia, Emotion regulation, Emotion beliefs, Self-help intervention, Online

## Abstract

**Objectives:**

Eating disorders (EDs) have a worldwide prevalence of 7.8%, with towering mortality rates and high healthcare costs. The current recommended treatment for EDs principally works by directly targeting ED thoughts and behaviours, but recovery rates are low. A multifaceted link between difficulties with emotions and EDs is now widely established, and newer third-wave therapies that aim to address these underlying emotion difficulties are promising. The current study piloted an online emotion self-help intervention which was co-developed with clinicians and people with lived experienced of EDs. The intervention aimed to specifically address difficulties with emotion identification and regulation, as well as unhelpful beliefs about emotions, which are believed to give rise to and maintain ED thoughts and behaviours.

**Method:**

We recruited 39 people with self-reported EDs to test this intervention over a one-week period. Our participants were asked to complete a series of questionnaires measuring emotion processes and psychopathology on Day 1 (T1) before being given access to the intervention. Participants were then asked to practice the newly acquired skills for seven days, before taking the same questionnaires on Day 9 (T2). We also asked participants to qualitatively report on their experience of the intervention.

**Results:**

We found significant improvements in ED psychopathology (ED-15), depression (PHQ-9), and anxiety (GAD-7) pre- to post-intervention, with medium to large effect sizes. All our emotion variables namely alexithymia (TAS-20), difficulties regulating emotions (DERS-SF), and unhelpful beliefs about emotions (EBQ) also showed significant changes post-intervention with medium to large effect sizes. Most importantly, changes in emotion regulation processes were linked to improved eating psychopathology. The qualitative analysis corroborated this finding, highlighting how the intervention helped them form new beliefs about emotions, which helped them reduce ED behaviours.

**Discussion:**

Significant improvements in emotion processing and regulations, as well as psychopathology, along with positive qualitative feedback, suggest that the intervention effectively met its aims of increasing awareness of the link between emotions and eating psychopathology, providing help to identify and regulate emotions, and normalising emotional experiences. While our results are promising, further research is required to assess its effectiveness longer term and in clinical settings.

**Supplementary Information:**

The online version contains supplementary material available at 10.1186/s40337-024-00974-5.

## Abstract (plain language summary)

About one person dies every hour as a direct result of an eating disorder (ED), in part because of poor treatment outcomes. Current recommended treatments have a limited focus around emotion difficulties, such as the ability to identify emotions and regulate them, which are linked to ED thoughts and behaviours. With the help of clinicians and people with lived experience of EDs, we developed an online intervention that works on these emotion difficulties. We recruited 39 people with EDs to test this intervention over a one-week period and evaluated our results using quantitative and qualitative analyses. Comparing scores before and after the intervention, we found that ED psychopathology, depression and anxiety were significantly lower post-intervention. Beliefs about emotions as well as the ability to identify, describe and regulate emotions were also significantly improved post-intervention. Qualitative analysis showed that our intervention helped people feel better equipped to deal with their feelings in a healthier way, which helped reduce ED behaviours. One person even described the intervention as “life-changing”. While our results are promising, further research is required to assess our intervention in a clinical setting.

**Supplementary Information:**

The online version contains supplementary material available at 10.1186/s40337-024-00974-5.

## Introduction

Eating disorders (EDs) are serious mental health conditions with a rising incidence. They have among the highest morbidity and mortality rates of all psychiatric illnesses, with suicide a major cause of death [73, 81]. They typically have a chronic course, resulting in many years lived with disability, annual healthcare costs almost double those of the general population, and 3.3 million healthy life years lost annually [83]. The scale of this problem is startling—EDs are estimated to affect 7.8% of the worldwide population [31], though their true prevalence is likely higher and apparently increasing [81]: the last four years showed a 21% increase in referrals in south-east England alone [4]. In addition, psychological treatment for EDs is suboptimal. Using the current empirically-supported evidence-based treatment (i.e. Cognitive Behavioural Therapy, CBT), over 60% of patients with Bulimia Nervosa (BN) [52] and 50% of patients with Binge Eating Disorders (BED) [51] do not obtain complete abstinence from core eating disorder symptoms. Similarly, relapse rates are high, for example with up to 53% of patients with Anorexia Nervosa (AN) relapsing [45], further demonstrating the limitations of current evidence-based treatments. Part of the answer may lie in the fact that current evidence-based treatments principally focus on the symptoms themselves, rather than what is driving them. Some evidence is however accumulating regarding a third wave of treatment that focuses on emotions as an underlying cause for these symptoms, such as dialectical behavioural therapy (DBT, e.g. [11]). We developed an online self-paced intervention based on DBT that draws on a body of knowledge concerning the relationship between difficulties with emotions and eating psychopathology [48, 54, 59, 82, 91]. This paper reports the findings from the pilot study evaluating the intervention in a group of 39 people with an ED.

**Fig. 1:**
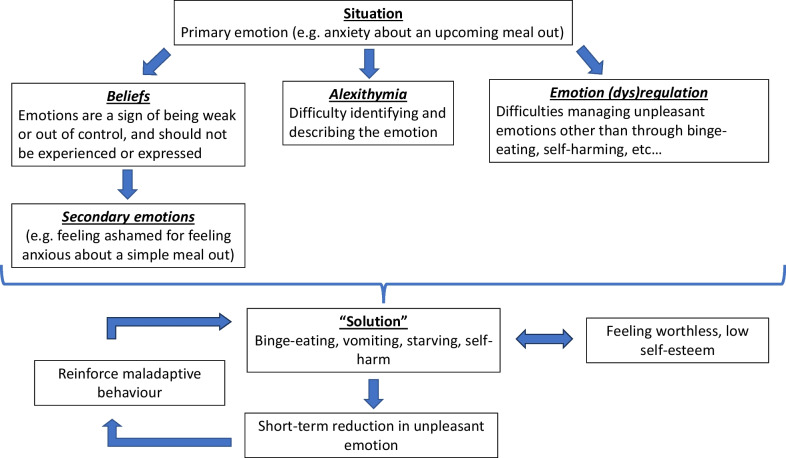
Beliefs, alexithymia and emotion regulation in eating disorders

A multifaceted link between difficulties with emotions and EDs is now widely established. Firstly, people with EDs struggle to *identify* and *describe* their emotions, a tendency known as alexithymia [91]. Secondly, they often struggle to *regulate* their emotions [64], seeming to over-rely on maladaptive emotion regulation strategies such as suppression or rumination while under-utilising adaptive strategies such as acceptance [64, 87, 88] or cognitive reappraisal [60]; indeed, lower use of cognitive reappraisal appears to be associated with higher severity of restrictive symptoms [86]. Thirdly, people with EDs also differ in their *beliefs* about emotions, such as whether emotions are controllable or uncontrollable, and whether they are good or bad which both have implications for emotion regulation strategies [30]. As such, people with EDs tend to believe that emotions are not something they can control or manage, with stronger beliefs around the uncontrollability of emotions being linked to greater ED psychopathology [87, 88], as well as higher levels of anxiety and depression [24]. The other dimension of beliefs about emotions concerns the extent to which they are good or bad, or perceived as acceptable or threatening. Maladaptive beliefs about the threat posed by emotions leads to emotional non-acceptance and can incur secondary emotions which have both been associated with EDs [23, 50]. While primary emotions are natural and adaptive responses to the environment (e.g. feeling sad when losing something or someone you care about), secondary emotions (e.g. feeling guilty for feeling sad because feeling sad is ‘bad’) are learnt responses which do not always make sense (e.g. the lingering emotion of *guilt* rather than sadness when losing something or someone you care about) and are known to be associated with ED behaviours [23]. Figure 1 summarises how each of these factors may contribute to ED behaviours.

There is evidence to support that at least some of these emotion difficulties, such as emotion regulation problems, are premorbid longitudinal predictors of disordered eating and EDs [38, 89]. Moreover, these concepts and difficulties are linked: for instance, being unable to identify emotions makes it harder to know how to regulate unpleasant feelings [16, 72], and believing emotions are uncontrollable influences the ways in which people attempt to regulate their emotions, if they try at all [30, 36]. However, these processes are also distinguishable. For example, alexithymia or maladaptive beliefs about emotions are not a prerequisite for difficulties with emotion regulations, which can happen at any and all stages of the emotion regulation cycle [35]. Therefore, we believe that interventions developed to help people with EDs better understand and manage their emotions should address all these concepts.

The foremost treatment recommended for EDs is Cognitive Behavioural Therapy [58], which principally works by directly targeting ED thoughts and behaviours [57]. However, newer therapies such as DBT aim to address underlying emotion difficulties, which are believed to give rise to and maintain ED thoughts and behaviours, as well as treating co-occurring psychopathologies (e.g. anxiety, depression) that also contribute to the development and maintenance of EDs [25]. DBT treatment focuses principally on emotional non-acceptance and offers strategies to help manage emotions. It has been shown to work well to improve emotion regulation skills and reduce eating psychopathology in EDs [69] and seems at least equally efficacious and less associated with relapse at 6-month follow-up compared to CBT [49]. However, it does not always resolve more fine-grained, underlying emotion processes, such as beliefs about emotions or alexithymia which can maintain ED behaviours and seem to act as a negative prognostic factor in ED recovery [74]. There is emerging evidence that interventions specifically targeting beliefs about emotions [33] or alexithymia [10] are effective for people with EDs and may lead to a higher probability of patients’ recovery [61]. As of yet, however, these emotion-focused approaches are not yet recognised or recommended by the National Institute for Health and Care Excellence (NICE); nor, to the best of our knowledge, has an intervention including *all* these significant aspects of emotional functioning, including beliefs about emotions, been developed.

Moreover, we do not know of an intervention that combine all these elements in an *online* platform, despite evidence showing that internet-based self-help approaches are effective to help reduce eating psychopathology [41, 68, 92, 93]. This is relevant because ED services are overstretched, with demand for treatment far outstripping capacity [76, 84]. Online self-help interventions offer the possibility to provide faster help to those waiting for treatment, and at potentially reduced cost [53]. With long waiting times translating into worsening of symptoms [85], higher total healthcare costs [14], and lower treatment uptake [29], providing treatment early without intensive clinical input could be an effective way to reduce the long waiting times [3].

The current study aims to evaluate a self-help online intervention that was developed by a team of clinicians and researchers, with input from lived-experience experts at multiple points. Through five short online self-paced videos (5–9 min long), it aims to increase awareness of the link between emotions and eating psychopathology, normalise emotions, and provide help to identify and regulate emotions, all the while using techniques known to improve therapeutic alliance through providing genuineness, unconditional positive regard, and empathic understanding [67]. We delivered this intervention to 39 people with EDs with the aim of answering the following research questions, using quantitative and qualitative methods:

1. Is our intervention linked with qualitative and/or quantifiable changes in emotion processes and psychopathology?
2. Do changes in emotional awareness and processing and regulating abilities predict changes in ED psychopathology?

## Methods

### Participants

Between May–July 2022, we used Prolific to recruit 39 participants with an ED who were living in the UK. We aimed for equal numbers of males and females but recruited 14 males and 25 females. Two female participants did not come back for the second part of the study, so the complete dataset includes 37 participants (n = 14 males). Most participants were cisgender, with one female identifying as transgender and another one as gender queer. All had a self-reported diagnosis of an ED (n = 7 with anorexia nervosa [AN]; n = 11 with bulimia nervosa [BN]; n = 13 with binge eating disorder [BED]; n = 5 with Eating Disorder Not Otherwise Specified [EDNOS] (equivalent to Other Specified Feeding and Eating Disorder [OSFED] in DSM-5); n = 1 Avoidant and Restrictive Food Intake Disorder [ARFID]). Twenty-seven participants said their ED was still active, and ten said they thought they were in remission. However, there was no difference in eating psychopathology scores between these two groups (M_ED active_ = 3.6, M_ED remission_ = 3.5, t(37) = 0.22, *p* = 0.831), so we included all participants. While we did not ask whether participants were presently taking any psychotropic medication or involved in any therapeutic psychological inventions, we asked at T2 whether they had started either since they began the study. No participants reported a change in psychological treatment but two reported a change in medication.

Mean age was 33.0 years (SD = 8.8, 20–56 range) and on average participants had been experiencing their ED for 4.9 years (SD = 7.1). The majority of our sample was White British (89.7%), with four participants identifying as Algerian (n = 1), Asian (n = 1), Black (n = 1) and mixed white and black Caribbean (n = 1). Some of our participants reported suffering from depression (n = 4), depression and anxiety combined (n = 10), OCD (n = 2), PTSD (n = 1), or agoraphobia (n = 1). Three participants reported a diagnosis of autism, one of dyslexia and one of attention deficit/hyperactivity disorder.

### Intervention development and detail

This intervention was based on models that consider difficulties in emotions to be a central trans-diagnostic phenomenon involved in the development and maintenance of EDs (e.g. [26, 50, 64]). As such, the first stage involved selecting specific emotional processes particularly relevant to EDs, based on theory as well as the clinical experience of the research team (two clinical psychologists working with patients with EDs -MGH and LM). We then developed and refined the videos and accompanying workbook and worksheets. This took the form of a two-stage iterative case series with n = 12 people with lived experience of EDs (n = 2 males; n = 6 AN; n = 3 EDNOS; N = 1 BN; n = 2 undisclosed). They were asked to comment on the aesthetic and EDI aspects of the intervention, as well as the content itself. This resulted in the intervention being tested in the current paper.

The current intervention takes the form of five animated videos developed using Videoscribe, each targeting a specific aspect of emotional difficulties (see Fig. 1), and consists of the following five modules:

- Video 1: Emotions have a purpose and are temporary (targeting *Beliefs about emotions*)
- Video 2: How to label emotions to reduce their impact (targeting *Alexithymia*)
- Video 3: Secondary emotions and their links with ED behaviours (targeting *Secondary emotions*)
- Video 4 and 5: Strategies to cope with emotions and lower emotional intensity (targeting *Emotion regulation*)

The intervention also incorporates a workbook summarising the content of the videos to improve accessibility (as suggested by the contributors with lived experience), and a booklet to support the practice of the newly acquired skills. The worksheet (provided on Day 1 and available throughout) allowed participants to add their reflection on each of the video, and provided DBT-type exercises to practice each new skills (identifying primary emotions; identifying secondary emotions; practicing cognitive reappraisal; identifying calming and pleasurable activities; practicing acceptance and letting go; and a final coping strategy mind map bringing in together all the newly learnt skills).

More details about the intervention including video links and workbook are available on request.

### Materials and procedure

**Fig. 2:**
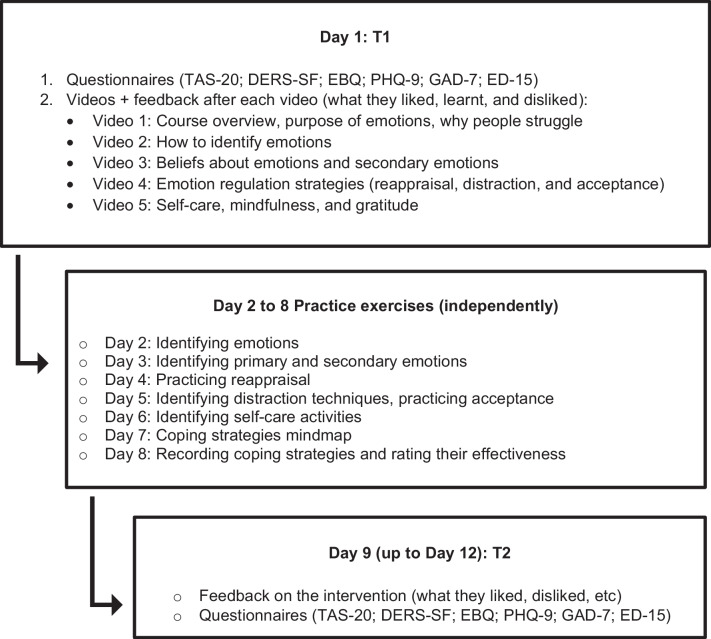
Flow diagram of the study

This study received ethical approval from the Research Ethics Panel at the lead author’s institution. The longitudinal design comprised two data collection sessions (Day 1, Day 9) interspersed by 7 days of self-completed practice, wherein no data collection occurred (see Fig. 2). Upon consenting to the study, participants completed the ‘Day 1’ session, which included completing the measures below, watching our five videos, and providing qualitative feedback on each. During this session, they were given access to our workbook and a summary document explaining the content of the videos and instructions for the days ahead Over Days 2–8, participants were guided through self-completion of 11 worksheets which helped them practice content related to the videos. Participants were told the research team were available for questions, although none contacted us to clarify the intervention or the exercises. The videos could be rewatched at any time as participants were provided with a YouTube link for easy access. After being reminded about it on Day 8, the Day 9 session became available to participants early that morning. This session involved providing feedback on the intervention and completing the same questionnaires as the Day 1 session. Participants were given 5 days to complete it: most (78%) completed it the day that it became available, but nine completed it on Day 10 and two on Day 12.

The questionnaires in order of completion on Days 1 and 9 are described below. All questionnaires showed good internal consistency (α > 0.70) with the exception of the externally orientated thinking (EOT) subscale of the Toronto Alexithymia Scale (TAS-20) (see Additional file 1). While this is consistent with previous reports of weak internal consistency for this subscale [19, 63], findings related to this construct must be interpreted with caution.

#### Measures of emotion processes

*The Toronto Alexithymia Scale (TAS-20):* This 20-item self-report measure [5] captures a three-faceted conceptualisation of alexithymia: identification of one’s own emotional states (difficulty identifying feelings: DIF), the ability to verbally describe emotional states to others (difficulty describing feelings: DDF), and an inclination away from introspection and towards externally orientated thinking (EOT). This three-factor model emerges robustly in analyses of the TAS-20 [71], which boasts strong psychometric properties [6]. Scores range from 20 to 100, with scores above 61 indicating clinically substantive levels of impairment (n = 17 participants were in that range at T1, and n = 10 at T2).

*The Difficulty in Emotion Regulation Scale, Short form (DERS-SF):* This 18-item self-report measure [44] assesses clinical impairments in emotion regulation. Its items correspond to six subscales: lack of emotional clarity, lack of emotional awareness, difficulties engaging in goal-directed behaviour when upset, difficulties with impulse control when upset, non-acceptance of emotions, and limited access to emotion regulation strategies (henceforth ‘clarity’, ‘awareness’, ‘goals’, ‘impulse’, ‘non-acceptance, ‘strategies’). This short-form of the original parent scale boasts strong construct validity, internal consistency and discriminative ability [18], outperforming other short forms of this scale [21]. Scores range from 18 to 90, with higher scores indicating more difficulties.

*Emotion Beliefs Questionnaire (EBQ):* This 16-item measure [9]

assesses two main categories of beliefs about negative and positive emotions: beliefs about their controllability and beliefs about their usefulness. The scale has three composite scores (total score, controllability, and usefulness), with each scale ranging from 8 to 56, and higher scores indicating more maladaptive beliefs about emotions (e.g., stronger beliefs that emotions are uncontrollable and not useful). It has good psychometrics properties [8], and although it has not been tested in a clinical population, it showed good internal reliability in our sample (see Additional file 1).

#### Measures of psychopathology

*The Patient Health Questionnaire-9 (PHQ-9) modified:* The PHQ-9 [47] is a brief, popular and well-validated [7] measure of depressive symptomatology, scoring from 0 to 27. With higher scores reflecting more severe depression, scores of 5–9, 10–14, 15–19 and 20–27 are indicative of mild, moderate, moderately severe and severe depression respectively. At T1, n = 31 participants reported depressive symptoms (n = 7 mild; n = 9 moderate; n = 7 moderately severe; n = 7 severe). At T2, n = 22 participants reported depressive symptoms (n = 10 mild; n = 10 moderate; n = 2 moderately severe; no participants reported severe depressive symptoms). While the scale typically assesses symptoms over the preceding 2-week period, we modified this at both data collection points to assess symptoms only over the previous week.

*The Generalised Anxiety Disorder-7 (GAD-7)* modified*:* The GAD-7 [75] is well-validated and in popular clinical use [62]. Scores range from 0 to 21, with scores of 5–9, 10–14 and 15 > being indicative of mild, moderate and severe anxiety respectively. At T1, n = 30 participants reported symptoms of anxiety (n = 9 mild; n = 11 moderate; n = 10 severe). At T2, n = 22 participants reported depressive symptoms (n = 10 mild; n = 10 moderate; n = 2 severe). As per the PHQ-9, we modified the GAD-7 at both data collection points to assess symptomatology over the previous 1 rather than 2 weeks.

*ED-15:* The ED-15 [77] was designed not as a screening tool but as a measure to reveal short-term changes in eating attitudes and behaviours. Ten items indicate past-week attitudes towards eating, weight and shape, while five (unscored) items indicate the past-week frequency of binge-eating, vomiting, restricting, laxative and exercise for purposes of weight loss. The attitudinal items which comprise its total score also yield two subscales, ‘Eating Concerns’ and ‘Weight and Shape Concerns’. For the total and the subscales, scores (computed by averaging across items) can range from 0 to 6, with higher scores indicative of greater psychopathology. The ED-15 possesses good psychometric properties and convergent validity with other validated measures of ED symptoms [56, 66].

#### Qualitative questions

Participants were asked open-ended questions about their experiences of the intervention. At T1, participants were asked two questions after watching each video which focused on obtaining feedback about the content. At T2, participants were asked seven open-ended questions, focusing on perceived impacts and the usefulness of strategies discussed in the intervention, as well as feedback about the course as a whole (see Additional file 1).

### Missing data and assumptions

There was no missing data in the complete dataset of 37 participants. All assumptions were checked. We had two outliers on the difference scores for the PHQ-9 (1 outlier) and the impulsive subscale of the DERS (1 outlier) which affected the skewness and kurtosis of these scales, but the results did not change after removing them from the analysis, so we kept them.

### Data-analysis

#### RQ1

Is our intervention linked with qualitative and/or quantifiable changes in emotion processes and psychopathology?

RQ1 utilised a mixed methods approach. Firstly, we conducted multiple paired sample t-tests on 19 variables: the total TAS-20 and three subscales (DIF, DDF and EOT), the total DERS and six subscales (strategies, non-acceptance, impulse control, goals, awareness and clarity), the total emotion beliefs and two subscales (controllability and usefulness), the PHQ-9, the GAD-7, and the ED-15 total and two subscales (Eating Concerns and Weight and Shape Concerns). Each variable was compared at T1 (before the intervention) and T2 (after a week). Effect sizes were calculated using Cohen’s d, the standardized mean difference between two groups. We also ran Wilcoxon t-tests to observe the difference in ED behaviours (bingeing, vomiting, laxative use, dieting and excessive exercise) at T1 (before the intervention) and T2 (after a week) given these variables were not normally distributed. Effect sizes were calculated using Wilcoxon’s r. We controlled for multiple comparison using Bonferroni correction, lowering the *p* value to *p* = 0.0021 to account for all 24 comparisons.

Secondly, we performed a thematic analysis of participant’s qualitative responses to explore their experiences of the intervention, using a data-driven approach. Two researchers analysed the qualitative data (MGH and CVT). Qualitative data were imported into NVivo 12 software, retaining the original punctuation, spelling mistakes and grammatical errors. Thematic analysis was then carried out using Braun and Clarke’s [15] six stage process: (1) data familiarisation, (2) data coding, (3) initial theme generation, (4) developing and reviewing themes, (5) refining, defining and naming themes, (6) writing up thematic analysis. MGH and CVT began by reading participant responses to support an in-depth familiarisation with the data. CVT then carried out initial coding for qualitative questions from T1 (feedback after each video) while MGH coded T2 data (perceived impacts and feedback from the intervention as a whole). CVT and MGH both came up with some initial theme ideas independently and then worked together to integrate and refine, working collaboratively to produce a coding document and thematic map. While the qualitative questions also focused on other aspects of the intervention such as barriers, facilitators and areas for development the qualitative analysis focused on experience and impact of the intervention itself, using the following questions to guide analysis: How was the intervention experienced? What are the processes through which the intervention was having an impact?. Themes were then presented to the entire research team, who provided critical feedback. Themes were then refined and written up by CVT and MGH. Figure 4 in the result section outlines the thematic map of themes and subthemes.

#### RQ2

Do changes in emotional awareness and processing and regulating abilities predict changes in ED psychopathology?

We ran a linear regression model, to determine whether changes in emotional knowledge and ability predicted changes in eating psychopathology. Our three predictors were conceptualised as difference scores between T1 and T2: the TAS total difference score to reflect improvements in difficulties identifying and describing emotions; the DERS-SF total difference score to reflect reduction in emotion dysregulation, and the EBQ total difference score to reflect improvements in the beliefs around the controllability and usefulness of emotions. Our outcome variable was the ED-15 total difference score between T1 and T2. We checked for assumptions which were all met, including the lack of multicollinearity between the variables (VIF scores below 5 and Tolerance above 0.1).

## Results

### RQ1

Is our intervention linked with qualitative and/or quantifiable changes in emotion processes and psychopathology?

**Fig. 3:**
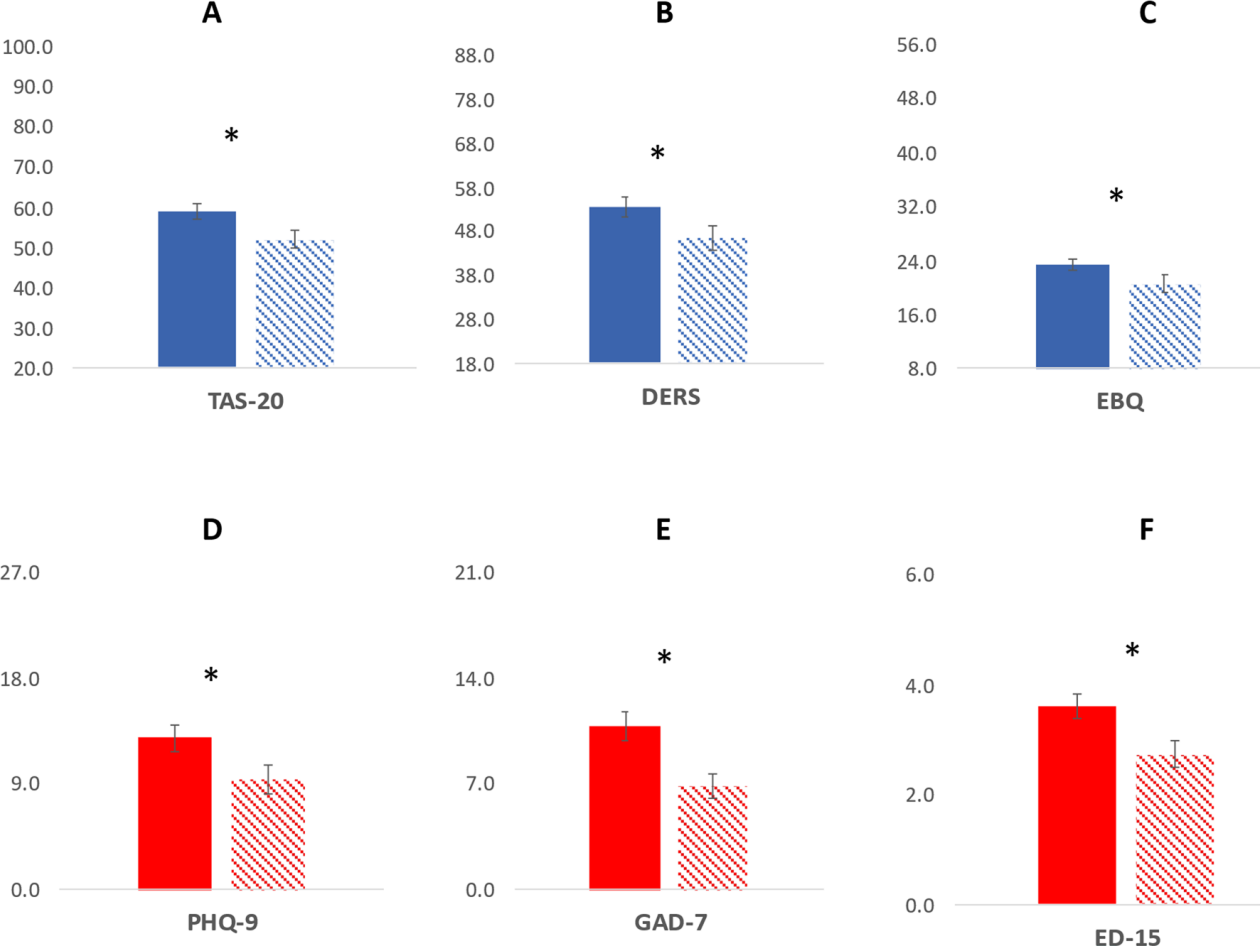
Bar charts representing key variables at T1 and T2

As shown in Table 1 and Fig. 3, there was a significant difference in most variables between T1 and T2. As such, the intervention influenced alexithymia (particularly difficulties identifying and describing emotions), emotion regulation difficulties (particularly non-acceptance of emotions, impulse control difficulties and clarity of emotions), as well as beliefs about the controllability of emotions. There was also a large significant reduction in eating psychopathology as well as depression and anxiety between T1 and T2. The intervention marginally reduced some ED behaviours such as binge-eating and dieting, although it did not reach significance after controlling for multiple comparisons.

**Table 1:** Comparisons of variables of interest at T1 and T2

	T1 Mean (SD), *range*	T2 Mean (SD), *range*	T-test *t*(df), *p*, [95% CI]	Effect size Cohen’s d or r
TAS	58.4 (12.5), *29–81*	51.9 (13.3), *24–76*	*t*(36) = 4.3, *p* < .001, [3.4 9.6]*	*d* = 0.70
DIF	22.6 (6.7), *7–34*	18.9 (7.3), *7–32*	*t*(36) = 4.8, *p* < .001, [2.1 5.2]*	*d* = *0*.*79*
DDF	16.1 (5.0), *5–25*	14.2 (4.4), *6–24*	*t*(36) = 3.2, *p* = 0.002, [0.6 3.0]*	*d* = *0*.*52*
EOT	19.8 (4.1), *12–27*	18.7 (4.8), 9*–27*	*t*(36) = 1.9, *p* = 0.035, [− 0.1 2.1]	*d* = *0.31*
DERS	53.6 (15.2), *22–82*	46.5 (15.7), *20–78*	*t*(36) = 3.6, *p* < .001, [3.1 11.0]*	*d* = 0.59
Strategies	9.6 (3.5), *3–15*	8.5 (4.0), *3–15*	*t*(36) = 2.6, *p* = 0.006, [0.3 2.1]	*d* = *0.43*
Non-acceptance	9.8 (3.5), *3–15*	8.3 (3.4), *3–15*	*t*(36) = 3.5, *p* < .001, [0.7 2.5]*	*d* = *0*.*58*
Impulse	7.6 (3.6), *3–15*	6.4 (3.3), *3–14*	*t*(36) = 2.9, *p* = 0.003, [0.4 2.1]	*d* = *0.48*
Goals	11.2 (3.4), *3–15*	9.8 (3.3), *3–15*	*t*(36) = 2.6, *p* = 0.007, [0.3 2.6]	*d* = *0.43*
Awareness	7.1 (2.6), *3–13*	6.7 (2.6), *3–13*	*t*(36) = 1.0, *p* = 0.162, [− 0.4 1.1]	*d* = *0*.*16*
Clarity	8.1 (3.0), *3–15*	6.9 (2.7), *3–12*	*t*(36) = 3.2, *p* = 0.002, [0.4 2.0]*	*d* = *0.52*
EBQ	23.3 (5.9), *12–38*	20.6 (7.2),* 9–39*	*t*(36) = 3.6, *p* < .001, [1.1 4.2]*	*d* = *0*.59
Controllability	25.9 (7.7), 8–40	21.4 (8.4), *8–42*	*t*(36) = 4.3, *p* < .001, [2.4 6.6]*	*d* = *0.71*
Usefulness	20.6 (5.9), 9–36	19.8 (8.2), *8–44*	*t*(36) = 1.08, *p* = 0.153, [− 0.8 2.4]	*d* = *0.17*
PHQ-9	12.8 (7.2), 0–27	9.4 (7.0), 0–27	*t*(36) = 4.2, *p* < .001, [1.7 5.0]*	*d* = *0.69*
GAD-7	10.8 (6.1), *0–21*	6.9 (5.1), *0–21*	*t*(36) = 5.8, *p* < .001, [2.5 5.2]*	*d* = *0.95*
ED-15	3.6 (1.3), *0–6*	2.7 (1.4), *0–6*	*t*(36) = 5.3, *p* < .001, [0.6 1.3]*	*d* = *0*.87
Weight concern	3.7 (1.6), *0–6*	2.7 (1.6), *0–6*	*t*(36) = 5.21, *p* < .001, [0.6 1.5]*	*d* = *0.86*
Eating concern	3.5 (1.2), *0–6*	2.8 (1.3), *0–6*	*t*(36) = 4.3, *p* < .001, [0.4 1.1]*	*d* = *0.70*
Bingeing	2.5 (2.7) 0–10	2.0 (2.9) 0–14	*z* = *− 2.1, p* = *0.033*	*r* = *− 0.35*
Vomiting	1.4 (4.0) 0–20	1.1 (3.6) 0–5	*z* = *− 1.3, p* = *0.190*	*r* = *− 0.21*
Laxative	0.5 (1.4) 0–7	0.4 (1.1) 0–7	*z* = *− 1.4, p* = *0.157*	*r* = *− 0.23*
Dieting	3.3 (2.5) 0–7	2.5 (2.3) 0–7	*z* = *− 2.0, p* = *0.049*	*r* = *− 0.33*
Exercise	2.0 (2.1) 0–14	1.7 (1.8) 0–6	*z* = *− 1.1, p* = *0.275*	*r* = *− 0.18*

The sections in italic represent the four emotional factors targetted in this online intervention

*p significant after Bonferroni correction (at *p* = .0021 to control for 24 comparisons)

Each graph represents one of the 6 variables. In blue (top row) are the three emotion variables (A) TAS-20, (B) DERS, (C) BES. In red (bottom row) are the three psychopathology variables: (D) PHQ-9, (E) GAD-7, and (F) ED-15. For each variable, average score at Time 1 is depicted in full bars, while average score at Time 2 is depicted in dashed bars. Standard error bars are represented and the * sign represents statistically significant differences between Time 2 and Time 2 at *p* < 0.001. Note that each graph has its own scale with min-max possible scores for each of the questionnaires.

**Fig. 4:**
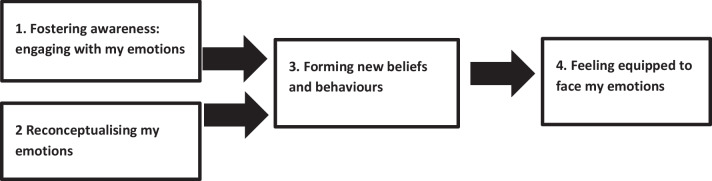
Thematic map of themes and subthemes

The qualitative analysis generated four themes from the data, as per Fig. 4 below: (1) Fostering awareness: engaging with my emotions, (2) Reconceptualising my emotions, (3) Forming new beliefs and behaviours, and: (4) Feeling equipped to face my emotions. These themes highlight the perceived mechanisms of the intervention and associated outcomes. Participant number, sex and ED diagnosis are included alongside illustrative quotes in the following section.

1. Fostering awareness: engaging with my emotions

Participants explained that the course fostered an increased awareness of their emotions. It was common for participants to state that they previously ignored, dismissed, or paid little attention to emotions. However, as a result of the intervention participants described noticing their emotions more frequently and being better attuned to what they were feeling and why.

I normally do not pay much attention to what specific emotions I am feeling- throughout the week I was being more mindful and attentive to what I was feeling rather than just ignoring my emotions. (P2, female, BED)

Consequently, some participants described having a newfound appreciation for the importance of emotions and recognising their emotional triggers.

It has made me more aware of what my emotions are and how they impact me. It has, for example, made me more conscious of getting enough sleep to help me with my emotions. (P9, male, BN)

Participants expressed that the course helped them to make better sense of their personal experiences, particularly through identifying and recognising secondary emotions. For example, one participant noted the value of logging and mapping their emotions:

I really found keeping a log useful as well as mapping. It was soothing to get thoughts down on paper and out of my head so I could work through them logically. This heed me to stop self sabbotaging and recognise whether I was experiencing secondary emotions or not. It also helped me to identify triggers for negative emotions. (P46, female, BED)

The course was perceived to be fundamental for many participants in helping them to notice and understand their emotional experiences.

2. Reconceptualising my emotions

Participants recognised that a key benefit of the course was that it encouraged them to reconceptualise their emotions. The course appeared to normalise emotions, helping participants realise that emotions were natural, transient, functional, and purposeful. In turn, this learning appeared to alleviate judgement and self-criticism towards emotional responses.

Normally if I felt a negative emotion I sort of assumed it was coming from inside me and was my fault, but being able to recognise them as natural responses to stimuli has made it easier to separate… the things I'm feeling from my self-worth. (P14, female, AN)

Many participants discussed the usefulness of learning to deconstruct their emotions, noting the importance of separating primary and secondary emotions as this allowed for emotional experiences to be better understood.

…Normally I find my emotions difficult to deal with as I seem to feel a lot of things that don't make (immediate) sense. Learning how to deconstruct my emotions and separate primary and secondary emotions helped to make more sense of what I was feeling. This was very useful to me as I stopped dismissing my emotions as stupid and illogical…. (P21, female, EDNOS)

By reconceptualising their emotions, some participants described newly understanding their ED behaviours as a means of coping with negative emotions, thus encouraging more adaptive responses through enhanced understanding of emotions.

I can recognise explicitly that unhealthy behaviours are being used as a direct way to cope with negative emotions. E.g: if I'm feeling sad i have a very strong urge to restrict my food, and I had never actually made the direct link that that was a way of trying to cope with the emotion. Being aware of this can help try and change to respond in healthier ways. (P14, female, AN)

Normalising emotions, as well as recognising primary and secondary emotions, helped participants to appraise their emotional experiences as valid and acceptable.

3. Forming new beliefs and behaviours

Participants also expressed the usefulness of the course in providing them with tools to engage in more adaptive coping behaviours and in helping them to form more accepting or helpful beliefs about emotions. Participants discussed employing the coping strategies provided in the materials, usually choosing one or two that particularly helped for them.

I feel I can now use this strategy to work on my current problems and like a weight is lifted to help unhook me from anchoring myself in past problems that seem to root me from moving forwards in life. This is wonderful and I cant wait to apply this to my own issues and see how I improve. These videos are an absolute god send to me and I am sure anyone who sees them. (P18, male, BED)

Many participants found practising acceptance in particular was key in helping them to engage with their emotional experiences more openly, rather than trying to suppress or dismiss their experiences.

The course also helped me to be less judgemental about my own emotions; I feel less guilty now when I am upset or angry, as I realise the guilt is an unhelpful secondary emotion, and that its okay to feel negative emotions from time to time. (P20, female, BED)

Participants described taking pause, reassessing their emotional experiences, and reappraising situations to diffuse difficult emotional experiences.

Reappraisal was particularly useful as well, I tried to use it when I felt annoyed or angry and it helped calm me down and let me see things in perspective. (P9, male, BN)

However, a small number of participants felt the intervention did not provide them with useful strategies to regulate their emotions. A small number of participants also described falling into a difficult pattern of overthinking, getting stuck in ruminative loops as a result of trying to rethink or re-appraise situations.

I have always struggled with reappraisal because my mind feels like it gets tied in knots and jams up with questioning everything, and doesn't know where to stop, and I end up not having a clue how I feel or think about anything and just being exhausted. (P29, female, BN)

Nevertheless, many participants reported that the course helped them to try out new behaviours and develop new self-awareness and understanding of their emotional world.

4. Feeling equipped to face my emotions

Participants recognised that the course allowed them to feel better equipped to face their emotions and find emotional balance; the skills they learned enabled them to feel more in control and less overwhelmed by, or reactive to, their emotions.

It gave me some distance and perspective about how I feel, and probably helped to control some overreactions I would usually have facing a wave of complex emotions, because I felt better equipped to face it. (P52, female, BN)

As a result, participants expressed a new sense of agency in being able to cope with their emotional experiences, proactively applying the tools they had learned from the course.

I also found focussing on self-care and breaking self-care down into different needs i.e., calm, pleasurable or achievement highly useful. This really helped me in taking the power and initiative to decide what I needed in moments of distress through understanding my emotions better which led me to feel far more in control of my emotions than I usually do, whilst also accepting them and understanding why they were coming up for me. (P24, female, EDNOS)

Feeling equipped to manage emotions had far-reaching consequences for some, significantly improving their quality of life and ability to cope.

A true life changing event I will take with me on the journey of life. This course is like a best friend that is always with you. …Its changed my life and helped me to heal. I have already found I am worrying much less. Sleeping much better. Getting on with things without and blocks of procrastination worry and lack of energy. I feel more present in the moment and less worried about the future or past. I try to keep calm and find solutions to challenges and see the past as what it is in the past but take positives from it now. I find I am more confident too and less withdrawn and more sociable. (P18, male, BED)

The impact of the course went beyond emotional experience for some participants, by also reducing ED behaviours. For example, in managing their emotions, participants described less purging, restricting and self-harming on a few occasions.

I'm responding to my emotions in a more patient manner as well as not engaging in unhealthy coping mechanisms the past week such as eating less or self-harming. I feel that I have found alternative ways to cope that actually feel just as effective and make far more sense. (P24, female, EDNOS)

Due to being more engaged with my emotions, I managed to stop myself from purging on a few occasions. (P51, male, BN)

Despite the course not focusing directly on eating behaviours, some participants also mentioned that the emotion regulation skills had helped them to reduce episodes of binge eating.

I don't normally take the time to fully appreciate or examine my emotions- the workbook encouraged me to do this which helped me feel much more relaxed, less stressed, and less likely to binge eat. (P20, female, BED)

Thus, it seemed that for many participants, the course provided skills that helped foster feelings of emotional balance, agency and control, as well as helping to manage ED behaviours for some.

### RQ2

Do changes in emotional awareness and processing and regulating abilities predict changes in ED psychopathology?

While the model significantly explained 21% of the variance in changes in eating psychopathology (adj. R^2^ = 0.21, F(3, 36) = 4.19, *p* = 0.013), only reduction in emotion dysregulation was a significant predictor (β = 0.50, *p* = 0.019), with improvement in alexithymia (β = − 0.10, *p* = 0.625) and beliefs about emotion (β = 0.17, *p* = 0.316) not significantly associated with changes in ED psychopathology.

## Discussion

This study piloted an online emotion self-help intervention which was co-produced with clinicians working with people with EDs (n = 2) and co-developed with people with lived experienced of EDs (n = 12). We recruited 39 people with EDs to test this intervention over a one-week period and evaluated our results using quantitative and qualitative analyses. Cautious interpretation of this pilot study is necessary given the relatively small sample, the absence of a control group, and the short follow-up period. Despite this, several findings indicate that the intervention may be a promising avenue for further investigation and development. Firstly, the intervention was completed by 37 of 39 participants: this, and the qualitative data, suggest it was feasible, engaging and acceptable to people with EDs. Secondly, we observed significant changes in ED psychopathology, depression and anxiety pre- and post-intervention, with medium to large effect sizes. All of our emotion variables including alexithymia, difficulties regulating emotions, and unhelpful beliefs about emotions showed significant changes post-intervention. Moreover, changes in emotion regulation processes were linked to improved eating psychopathology. The qualitative analysis corroborated this finding in more depth, suggesting that the intervention helped people form new beliefs about emotions which made them feel equipped to deal with their feelings in a healthier way, leading to a perceived reduction in ED behaviours. Indeed, while we did not directly ask participants if they perceived a change in their ED symptoms and, if so, their interpretation of that effect, participants did volunteer that they had successfully circumvented self-injurious and ED behaviours directly through practice of emotion regulation skills.

There is precedent for the efficacy of emotion-focused interventions in EDs, and different approaches corroborate our findings. For instance, Holmqvist Larsson et al. [39, 40] delivered their emotion regulation skills training in the form of five two-hour group sessions with homework. At the last session, their 29 participants reported alleviation of alexithymia, difficulties with emotion regulation, depression and ED psychopathology, with reductions in emotion regulation difficulties correlating with reduced clinical impairment related to EDs. Berking and colleagues [12] similarly adopted an in-person group approach for their affect regulation training regime, and found significant reductions in binge eating and general ED psychopathology which surpassed changes observed in a waitlist comparison group. Much like our participants, other researchers found that people with EDs perceive emotion-focused approaches as beneficial [32, 39, 40, 78]. This is also indirectly corroborated by studies which demonstrate the efficacy of DBT in addressing alexithymia, emotion regulation difficulties, and ED psychopathology [11, 65]. In particular, one found that DBT skills in emotion regulation, distress tolerance, mindfulness and interpersonal effectiveness mediated the relationship between reduced emotional dysregulation and reduced ED psychopathology [43].

While our paper adds to this growing literature and could address high rates of relapse by focusing on the underlying emotion difficulties which precede and maintain ED thoughts and behaviours [38, 80, 89], it also suggests that psychoeducation about emotions can be successfully conducted *online* in a cost-effective way. In the UK, demand for treatment far outstrips the capacity of adult ED services [76, 84]. This leaves the majority of individuals with EDs to suffer long waiting times [3], which translate into worsening of symptoms [85], higher total healthcare costs [14], lower treatment uptake [29], and less likelihood of responding positively to treatment [3, 27]. Whilst unlikely to be curative without any clinical input, online self-help interventions offer the possibility to provide help to those waiting for treatment at potentially reduced cost [53]. Current evidence supports the fact that internet-based self-help approaches are effective to help reduce eating psychopathology [41, 68, 92, 93], and we recommend future work should test whether our intervention could be successfully delivered whilst patients await face-to-face treatment.

### Strengths, limitations, and future directions

Our study has some clear strengths. First, we attempted to address the absence of cisgender men from the literature on EDs and clinical interventions [17, 22, 28]. Sex and gender are important moderators of ED experience and treatment needs [20, 46], and moreover appear to affect the inclination and/or ability of individuals with ED to utilise certain emotion regulation strategies [86]. While differences in the qualitative responses of men and women were not directly explored in our study, men and women might benefit differently from the components of the intervention, and future research should explore this quantitatively as well as qualitatively.

Secondly, we attempted to ensure diversity in the spectrum of ED psychopathology, particularly with regards to the commonly overlooked ED of BN, BED, and EDNOS/OSFED [1]. With only 7 participants with AN, 11 with BN, 13 with BED, and 5 with EDNOS, our study was however unfortunately underpowered to compare diagnostic categories (or, moreover, to examine their potential interactions with sex/gender). The range of diagnoses also most likely prevented us from seeing a change in ED *behaviours* such as binge-eating because not all participants engaged in these behaviours at T1. We consider it likely however that the intervention might be particularly beneficial for people with BN, BED and certain types of OSFED, due to the particular link between emotion dysregulation and binge eating as well as purging [2, 12, 90]. Whilst emotion difficulties seem to be a transdiagnostic factor not distinguishing between the various EDs [64], dysfunctional emotion regulation seems related with different eating outcomes in AN vs BN [55]. Moreover, different types of DBT have been suggested to work best for AN vs BN/BED [11]. For example, people with AN tend to be overcontrolled with their emotions and as such may beneficiate more from radically open DBT (RO-DBT) which focuses more on increasing an individual's awareness of social signalling [37]. BN and BED however tend to be more emotionally undercontrolled and DBT, which works more on people’s ability to tolerate distress and reduce impulsivity—and on which our intervention was based—seems to work better for these diagnoses [70, 79]. While our qualitative study suggested that participants with AN also found the intervention helpful, it is likely that personalisation of the intervention might improve its efficacy for individuals with different types of ED. Some patients with AN may also require nutritional rehabilitation first to benefit [42]. This, indeed, highlights further questions regarding optimal timing for delivery; while it could be helpful to be delivered while patients with EDs wait for treatment, it could also be delivered as an add-on to current treatment once individuals have stabilised their eating behaviours.

There are a number of limitations to the present study. First, lacking the control of a randomised clinical trial and without any control comparison groups, we are unable to confidently attribute observed changes to the intervention rather than other factors such as positive feelings about contributing to research, expectancy effects, social desirability, or other changes in circumstances unrelated to the intervention. We did ask about changes in life circumstances between T1 and T2, and only two reported a change in medication, which is unlikely to have had any effect given the short lapse between the two time-point (1 week). Relatedly, we also asked for qualitative feedback at T2 before the quantitative assessment, which may have influenced the results. Second, while we hypothesised a causal pathway where ED psychopathology might be alleviated through improved emotion processing, our ability to assess the mechanism of this intervention was limited. Ideally, such a design should include at least three timepoints of measurement, although more numerous timepoints are informative in revealing effects that are immediate and constant, immediate but declining, or effects that exponentially or linearly develop [13, 34]. We have no way of knowing, indeed, whether effects 1-week post-intervention were stable. However, our finding that reduced emotion regulation difficulties predicted reduced ED symptomatology is consistent with the relationship that Holmqvist Larsson and colleagues [39, 40] observed between reduced difficulty with emotion regulation and reduced clinical impairment related to EDs; and with Berking and colleagues’ [12] finding that reduced BED symptoms was partially mediated by improvements in emotion regulation.

Finally, whilst we had only two dropouts, we are unaware of those individuals who elected not to participate, and did not explore characteristics which might affect treatment uptake and response in a real clinical setting. Our sample was, for instance, unrepresentative of neurodivergent, non-binary and/or transgender individuals with ED, and of ethnic/racial minorities. More broadly, it is probable that participants on Prolific are unrepresentative of individuals seeking or waiting for treatment from public health services, whose expectations and hopes for professional involvement might differ, if only just due to the financial incentive our participants received to take part in the current study. Indeed, while the financial incentive (around £12, or £7.5/hour) is unlikely to have influenced the findings themselves (i.e. participants were paid regardless of the quality or directionality of their responses), it may likely have increased engagement with the study (i.e. participants were paid separately for time point 1 and 2). As such, it is imperative that it is tested in real-world settings without such incentives.

## Conclusion

In piloting our emotion-focused intervention co-produced with clinicians with inputs from people with lived experience, we observed significant reductions in depression, anxiety and ED psychopathology between time points 1 and 2. Significant improvements in identification and ability to describe emotions, emotion dysregulation, and beliefs in the controllability and usefulness of emotions, along with positive qualitative feedback, suggest that the intervention effectively met its aims of increasing awareness of the link between emotions and eating psychopathology, providing help to identify and regulate emotions, and normalising emotional experiences. While one participant described their participation as a “life-changing event”, another participant also reported struggling to understand how to use reappraisal in real life, so further research, with longitudinal, controlled designs need to continue improving the intervention and assess its suitability for people with different kinds of EDs in clinical settings.

### Supplementary Information

**Additional file 1. Table S1**: Scale internal consistency at Time 1 (T1) and Time 2 (T2).

## Data Availability

The datasets used and/or analysed during the current study as well as the intervention materials are available from the corresponding author on reasonable request.

## Abbreviations

AN: Anorexia nervosa
ARFID: Avoidant/restrictive food intake disorder
BED: Binge eating disorder
BN: Bulimia nervosa
CBT: Cognitive behaviour therapy
DBT: Dialectical behavioural therapy
DERS: Difficulties in Emotion Regulation Questionnaire
EBQ: Emotion Beliefs Questionnaire
ED: Eating disorder
EDNOS: Eating disorder not otherwise specified
ED-15: Eating Disorder Examination Questionnaire
GAD-7: Generalised anxiety disorder-7
NHS: National Health Service
OSFED: Other specified feeding and eating disorder
PHQ-9: Patient Health Questionnaire -9
RO-DBT: Radically open dialectical behavioural therapy
TAS-20: Toronto alexithymia scale-20
