# Supplementary material for: Early evaluation of a DBT-informed online intervention for people with eating disorders
Source: J Eat Disord. 2024 Jan 19;12:9. doi: 10.1186/s40337-024-00974-5 (PMC10799469; doi:10.1186/s40337-024-00974-5)
Supplement: Supplementary file 1 — Additional file 1. Table S1: Scale internal consistency at Time 1 (T1) and Time 2 (T2). [file 40337_2024_974_MOESM1_ESM.docx]

**Supplementary materials**

| Measure | α at T1 | α at T2 |
| --- | --- | --- |
| TAS-20 total  DIF  DDF  EOT | .85  .84  .85  .48 | .88  .90  .77  .69 |
| DERS total  Clarity  Awareness  Goals  Impulse  Non-acceptance  Strategies | .94  .86  .81  .92  .95  .91  .89 | .95  .88  .82  .92  .93  .88  .94 |
| Beliefs  Controllability  Usefulness | .82  .66 | .85  .81 |
| PHQ-9 | .92 | .93 |
| GAD-7 | .94 | .91 |
| ED-15  Weight concern  Eating concern | .93  .96  .73 | .94  .94  .80 |

1. **Scale internal consistency at Time 1 (T1) and Time 2 (T2)**
2. **List of qualitative questions at T1 and T2**

Time 1 (repeated after each five videos):

- Can you please tell us what you liked and what you learnt from the video?
- Can you please tell us if there is something you did not like or that was not clear?

Time 2 (asked once):

- Thinking about this online course: What did we do well?
- Thinking about this online course: What could we do better?
- Did you find you were thinking more about your emotions over the course of the week?
- What impact has this online course had on how you *think*about your emotions?
- What impact has this online course had on how you *respond to and cope* with your emotions?
- Were there any particular strategies from the videos that you found useful? If so, in what way?
- Were there any particular strategies from the videos that you found difficult? If so, in what way?
